# Developing Quality Indicators for the Pharmacological Management of Chronic Non-Cancer Pain in Older Adult Inpatients: A RAND/UCLA Delphi Study

**DOI:** 10.2147/JPR.S533027

**Published:** 2025-09-27

**Authors:** Aljoscha Noël Goetschi, Nicole Schönenberger, Ursina Wernli, Carla Meyer-Massetti

**Affiliations:** 1 Clinical Pharmacology and Toxicology, Department of General Internal Medicine, Bern University Hospital, Bern, Bern, Switzerland; 2 Graduate School for Health Sciences, University of Bern, Bern, Bern, Switzerland; 3 Institute of Primary Health Care (BIHAM), University of Bern, Bern, Bern, Switzerland

**Keywords:** chronic non-cancer pain, drug therapy, medication safety, medication review, older adults

## Abstract

**Purpose:**

Chronic non-cancer pain (CNCP) is a disabling condition affecting many older adult inpatients. While first-line therapy for CNCP consists of non-pharmacological approaches, many older adults receive pharmacotherapy nevertheless, putting them at a high risk of medication-related problems. Quality indicators (QIs) for the pharmacological management of CNCP could help reduce this risk. This Delphi study aimed to establish the face validity and feasibility of a list of previously developed QIs for the pharmacological management of CNCP in older adult inpatients.

**Patients and Methods:**

We followed the RAND/UCLA Delphi study methodology to establish an expert consensus on a list of proposed QIs. Over two written rounds, nursing, pharmacy and medical experts rated the face validity and feasibility of the QIs identified in a previous systematic literature search. QI ratings that were uncertain or disagreed upon after the first round were discussed in three expert focus group discussions. The QIs discussed were rated again in round two, and the most relevant QI in each category was prioritised.

**Results:**

Twenty-two experts agreed to participate in the study’s deliberations. Nineteen experts (86%) returned their ratings in each written round, and 9 (41%) participated in the focus groups. They evaluated 61 proposed QIs, modified 11 of them and suggested 13 new ones. The final set consisted of 51 QIs, with the experts prioritising 23 different ones. The 51 QIs covered the categories of general pharmacotherapy and the appropriate use of opioids, non-steroidal anti-inflammatory drugs (NSAIDs), paracetamol, metamizole and co-analgesics.

**Conclusion:**

Through consensus, we developed a first set of QIs for the pharmacological management of CNCP in older adult inpatients. This set will help standardise care, track and benchmark the quality of care, and be used as a trigger to prioritise patients for clinical or pharmacological interventions.

## Introduction

Chronic non-cancer pain (CNCP) significantly reduces the quality of life of the individuals affected by it. As 28–88% of older adults may suffer from CNCP, this is a highly relevant public health concern.^1^ CNCP is associated with multimorbidity in 88% of cases,^2^,^3^ with depression and insomnia being frequent co-morbidities.^4–6^ Older adults suffering from CNCP, in particular, have more physical and cognitive deficits.^7^ CNCP affects more women than men and more people from poorer socioeconomic backgrounds, particularly those with past unemployment.^8^,^9^ Alcohol consumption and smoking also seem to correlate with CNCP.^10^,^11^

Because of its complexity, CNCP management should consider different treatment types. First-line therapies should be non-pharmacological and can include both psychological and somatic interventions. Pharmacological therapies should be considered second-line therapies.^12^ Despite this recommendation, however, many older adult inpatients continue to be prescribed drugs for their CNCP as a first-line treatment, putting them at greater risk of medication-related problems (MRPs).^7^

MRPs are particularly prevalent among older adult inpatients, largely due to their complex clinical presentations involving multimorbidity and polypharmacy.^13^ As older adult patients often experience clinical decompensation during hospitalisation, the effects of MRPs are more detrimental.^14^ The situation becomes even more complex in the context of CNCP, because older adult patients may experience acute-on-chronic pain, which may require additional pharmacological treatment.^15^ Older adults continue to be at a very high risk of MRPs when they are discharged from hospital due to suboptimal information transfer and communication issues.^16^,^17^ These intersecting challenges highlight the importance of improving medication safety practices for older adult patients with CNCP.

Quality indicators (QIs) are measurable items used to assess and track care, both within and between institutions.^18^ As such, QIs can help to standardise healthcare processes and improve the quality of care delivered.^18^ If incorporated within (electronic) trigger tools, QIs can help to efficiently identify patients at a higher risk of deficient care.^19^ QIs are thus essential to continuous improvements in healthcare system quality and must be developed with great rigour.^18^,^20^

Although there are different methods for developing QIs, most involve systematic searches of the relevant literature and/or the expert validation of the QIs selected.^18^ Our previous systematic literature search revealed no existing set of QIs to guide the pharmacological management of CNCP in older adults, but we did accumulate a list of individual QIs and developed new ones from the quality criteria found.^21^ However, QIs must also reflect expert opinions,^18^ especially in a domain where high-quality evidence from randomised controlled trials is scarce.^21^ The Delphi method is a suitable study design for achieving expert consensus on QIs. It is a qualitative, constructivist, consensus-building method that allows experts to negotiate a shared clinical reality and co-construct recommendations and rules.^22^ However, regional differences in drug availability or guidelines may cause experts to rate QIs differently, limiting their generalisability. The present study aimed to find a consensus on the face validity and feasibility of a proposed set of QIs for the pharmacological management of CNCP in older adults inpatients.

## Materials and Methods

### Design

Campbell et al proposed that developing QIs first requires collating evidence using a systematic literature search and then establishing expert consensus on the QIs identified.^18^ To this end, they explicitly recommended using the Research and Development Corporation’s (RAND) and the University of California at Los Angeles’s (UCLA) Appropriateness Method, known as the RAM.^18^,^23^ Prior to our RAM Delphi study, we used an integrative literature review to systematically collect an initial set of potential QIs for the pharmacological management of CNCP in older adult inpatients.^21^

**Figure 1:**
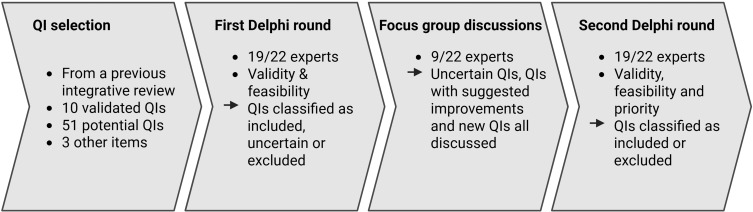
Schematic overview of the methods used in this RAND/UCLA Appropriateness Method Delphi study. QI = Quality indicator. Created in BioRender. Goetschi, A (2025) https://BioRender.com/i93a003.

To find a consensus on the face validity and feasibility of this proposed set of QIs, we conducted a two-round Delphi study with three focus group discussions between the two rounds, as the RAM proposes. In the first round, experts rated the face validity and feasibility of the proposed set of QIs. Those for which no consensus could be found or for which we received many suggestions for improvement were discussed in three focus groups. Each expert was asked to participate in one focus group. Subsequently, QIs without a consensus decision and those that had been adapted were rated again. A visual representation of the RAM Delphi study methods used is shown in Figure 1. For the study overall, we followed the Recommendations for the Conducting and Reporting of Delphi Studies (CREDES).^22^ Our local ethics committee (Cantonal Ethics Committee Bern) declared that the study did not require its approval (Req-2024-00779) because the project does not fall under the Human Research Act, Art. 2, para. 1.

### Expert Selection

The RAM recommends creating an expert panel of 7–15, but acknowledges that the optimal composition depends on the desired geographic and professional representation.^23^ As we aimed for a diverse and international panel including nurses, pharmacists and physicians, we set our target panel size at 20–30 experts. Experts were required to hold a university degree in nursing, pharmacy or medicine and have proven expertise (clinical or academic) in the pharmacological care of older adult inpatients with CNCP. Experts were mainly identified from the authors’ professional networks and from relevant published articles found in our systematic literature search.^21^ If experts recommended suitable candidates, we contacted them equally. We contacted potential experts via Email and re-contacted them two weeks later if they had failed to respond.

### Selection of QIs to Be Rated

We conducted a systematic literature search using an integrative review to screen 6,842 articles. This identified 11 validated QIs, 10 of which were included in the Delphi study.^21^ The QI excluded covered primary care practices and fell outside of our focus on inpatient care. Of the 243 other existing QIs identified, we included every QI mentioned in at least four independent studies or deemed of specific relevance by the research team, leading to a further 51 QIs being included in the Delphi study. Thus, our experts judged a total of 64 items in the first round (61 QIs plus three questions/clarifications).

### Written Delphi Rounds

Our RAM study used two written Delphi rounds to establish an expert consensus. In both rounds, the experts rated the face validity and feasibility of each item on a Likert scale ranging from 1–9 (1 = extremely invalid/unfeasible; 5 = uncertain; 9 = extremely valid/feasible). Experts were able to comment on each QI if they thought it necessary and could suggest new QIs. ANG set up both questionnaires in Excel^®^ (Microsoft^®^ Excel^®^ 2016 (16.0.5448.1000) MSO (16.0.5452.1000) 32 Bit) sheets (see Supplementary File 1) and they were pilot tested by three independent researchers: NS, UW and CMM. Both rounds also contained a disclaimer (see Supplementary File 1) stating that by participating in the study, experts agreed to further anonymous use of their data.

The experts rated 64 items in the first round, which took place from 10 to 31 June 2024. To facilitate experts’ decisions, we provided them with a synopsis of the results from the integrative review.^21^ We followed the RAM recommendations to define their consensus view:^23^

1. Include: a panel median score of ≥ 6.5, without disagreement in either face validity or feasibility.
2. Exclude: a panel median score of < 3.5 in either face validity or feasibility, without disagreement.
3. Uncertain: a panel median of ≥ 3.5 and < 6.5 in either face validity or feasibility, or disagreement. The “exclude” category had priority: if one category was “uncertain” and the other was “exclude”, then the QI was excluded.

We calculated disagreement using the Interpercentile Range Adjusted for Symmetry required for disagreement (IPRAS) proposed by the RAM.^23^

For the second Delphi round, which took place after the focus groups (described in the next paragraph), we applied the same procedures. Experts received an Excel spreadsheet showing the median first-round ratings for each QI, plus their own ratings. In addition, we asked the authors to list the most important QI in each pharmacological category (ie general drug therapy, opioids, NSAIDs, paracetamol, metamizole and co-analgesics). The second round took place from 14 to 28 August 2024 and involved rating the uncertain QIs again and rating the newly proposed QIs. We had decided a priori to limit the workload and time burdens put on the experts to two rounds of rating using the two questionnaires and participating in a 1.5-hour focus group. This approach overcame the possible limitations of a fixed two-round procedure, and we also excluded from the second round all the QIs that had not met the criteria for the “include” category.

### Focus Group Discussions

Semi-structured focus group discussions occurred between the two written Delphi rounds. These enabled our experts to exchange thoughts and supported the co-construction of a shared clinical reality, recommendations and rules.^22^,^23^ We held three 1.5-hour focus group discussions, on 5, 6 and 9 August 2024, to ensure the participation of as many experts as possible. Experts were strongly encouraged to attend one meeting. Experts failing to attend a meeting were not excluded from the second Delphi round, however, and they received a synopsis of the issues discussed. Indeed, all the experts received this synopsis to ensure that they were aware of the points discussed by all three focus groups.

We tried to balance the composition of these discussion meetings using profession as the main criterion. Meetings followed the RAM recommendations to ensure that each expert had an opportunity to speak up and give their perspective.^23^ The focus group discussions were semi-structured, meaning that they followed the same pattern and all covered the same relevant topics. Discussions nevertheless remained open to any expert input, and schedules were adjusted if we felt the participants needed to discuss an issue in greater depth. According to the RAM recommendations we ensured that all experts could voice their opinions.^23^ In the event of disagreement, we attempted to facilitate a productive debate. If no consensus could be reached, we raised the issue in the next focus groups. If there was still no consensus, we let the experts decide in the second written round. All the discussions were led by ANG, who is a 27-year-old male pharmacist and PhD candidate in clinical pharmacy. He works clinically on geriatric and internal medicine wards and has experience in facilitating focus group discussions. Either NS or CMM were present at every meeting and took notes *verbatim*. Meetings were held using Zoom (^©^2024 Zoom Video Communications, Inc). Prior to starting the meetings, all the experts gave their consent to participate and to the recording of the event. We used the recordings to verify and complete the notes and then deleted them.

## Results

### First Round

**Figure 2:**
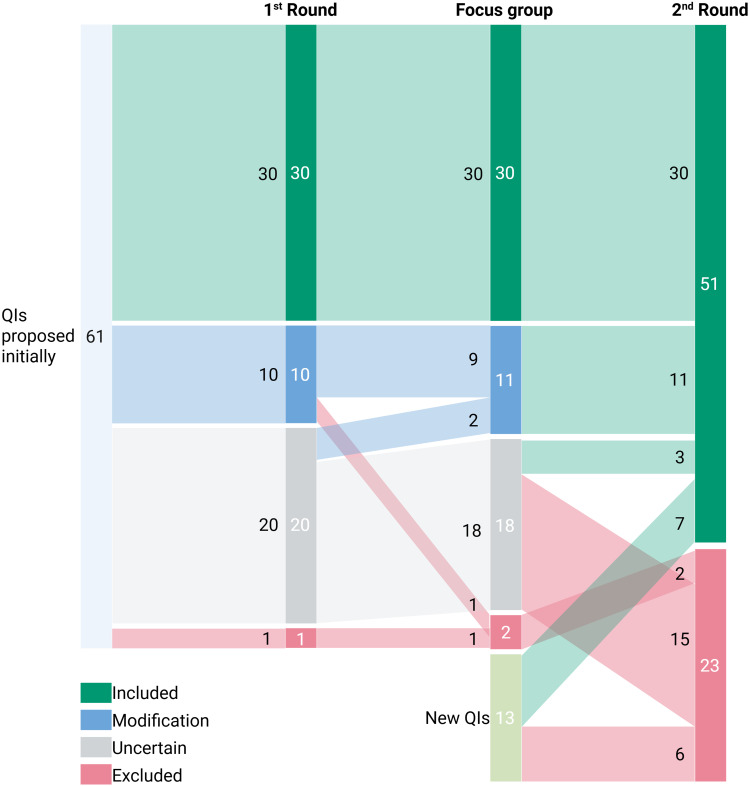
Sankey plot depicting how the experts evaluated, modified, rated uncertain, included and excluded the 61 original QIs identified in a previous integrative review, plus an extra 13 new QIs, over the two written rounds and the focus groups. Created in BioRender. Goetschi, A (2025) https://BioRender.com/m32m131.

We initially contacted 52 experts, and 22 agreed to participate. In the first Delphi round, 19 (86%) experts returned their ratings (see Table 1 for the experts’ characteristics). Of the 61 QIs rated, 30 had panel median scores ≥ 6.5 for both face validity and feasibility. Ten QIs that received suggestions for improvement, were retained and discussed in the ensuing focus groups. Twenty QIs had uncertain ratings, and one was excluded (see Table 2 and Figure 2).

**Table 1:** Summary of the Characteristics of the Experts Who Agreed to Participate in Our Delphi Study. As Some Experts Were Working in Multiple Settings, the Percentages and Absolute Numbers May Add up to More Than 22

Characteristic	Number of experts (n = 22)	
Gender	Female	16 (73%)
	Male	6 (27%)
	Other	0 (0%)
Profession	Pharmacist	11 (50%)
	Physician	6 (27%)
	Nurse	5 (23%)
Years of experience	> 20 years	9 (41%)
	16–20 years	6 (27%)
	11–15 years	1 (5%)
	6–10 years	5 (23%)
	1–5 years	1 (5%)
Work setting	Inpatient care	17 (77%)
	Research	10 (45%)
	Outpatient care	4 (18%)
	Long-term care	2 (9%)
Country of work	Switzerland	17 (77%)
	Germany	4 (18%)
	Qatar	1 (5%)

**Table 2:** Overview of the Median Expert Ratings for Face Validity and Feasibility, as Well as Disagreements Concerning Quality Indicators (QIs) Over the Two Delphi Rounds

No.	Quality Indicator	First Round					Second Round					New No.
		Validity		Feasibility		Decision	Validity		Feasibility		Final Decision	
		Median	Dis.^1^	Median	Dis.^1^		Median	Dis.^1^	Median	Dis.^1^		
1	IF an older adult has a new diagnosis of CNCP^2^, THEN pharmacological treatment should be provided.	6	No	8	No	Uncertain	7	No	n/a	n/a	Included	G5
2.1	IF an older adult is being treated for CNCP^2^, THEN they should be assessed for a response within 3–6 months.	8	No	7	No	Discussion	n/a	n/a	n/a	n/a	Excluded	
2.2	IF an older adult is being treated for CNCP^2^, THEN they should be assessed for a response within 1 month.	n/a	n/a	n/a	n/a	n/a	9	No	7	No	Included	G4
3.1	IF an older adult with CNCP^2^ presents with moderate to severe pain (score ≥ 6 on a scale of 0–10 or a similar quantifiable measure), THEN pain treatment should be adjusted if aligned with care goals.	8	No	8	No	Discussion	n/a	n/a	n/a	n/a	Excluded	
3.2	IF an older adult with CNCP^2^ presents with acute pain (score ≥ 6 on a scale of 0–10 or a similar quantifiable measure on an assessment tool for cognitively impaired patients), THEN pain treatment should be adjusted if aligned with care goals.	n/a	n/a	n/a	n/a	n/a	8	No	8	No	Included	G7
N1	IF an older adult has CNCP^2^, THEN define care goals with the patient that focus on quality of life and functionality.	n/a	n/a	n/a	n/a	n/a	9	No	7	No	Included	G1
4	IF an older adult with CNCP^2^ is treated using opioids, THEN they should be offered a bowel regimen or medical records should document the potential for constipation or explain why bowel treatment is not needed.	9	No	7.5	No	Included	n/a	n/a	n/a	n/a	Included	O6
5.1	IF an older adult with CNCP^2^ starts a new opioid therapy, THEN efficacy and side effects should be assessed after 1 week to 1 month.	9	No	7	No	Discussion	n/a	n/a	n/a	n/a	Excluded	
5.2	IF an older adult with CNCP^2^ starts a new opioid therapy or if the opioid dose is changed, the efficacy and side effects should be closely monitored and evaluated no later than 1 week after starting/changing therapy.	n/a	n/a	n/a	n/a	n/a	9	No	7	No	Included	O1
N2	IF an older adult with CNCP^2^ starts opioid therapy, THEN evaluate after no more than 3 months whether the therapy significantly improves the patient’s quality of life.	n/a	n/a	n/a	n/a	n/a	9	No	7	No	Included	O3
6	IF an older adult with CNCP^2^ requires analgesia, THEN pethidine/meperidine should not be used.	9	No	9	No	Included	n/a	n/a	n/a	n/a	Included	O7
7	IF an older adult has been prescribed a cyclooxygenase-nonselective NSAID^3^ for the treatment of CNCP^2^, THEN medical records should indicate whether they have a history of peptic ulcer disease and, if they do, justification of NSAID^3^ use should be documented.	9	No	7	No	Included	n/a	n/a	n/a	n/a	Included	N5
8.1	IF an older adult with CNCP^2^ is aged ≥ 75 or has a history of peptic ulcer disease or gastrointestinal bleeding or currently uses antithrombotics/anticoagulants AND they are treated using a cyclooxygenase-nonselective NSAID^3^, THEN they should be provided concomitant treatment with misoprostol or a proton pump inhibitor.	9	No	8	No	Discussion	n/a	n/a	n/a	n/a	Excluded	
8.2	IF an older adult with CNCP^2^ - has a history of peptic ulcer disease or gastrointestinal bleeding and/or - currently uses antithrombotics, anticoagulants, corticosteroids or SSRIs AND they are treated using a cyclooxygenase-nonselective NSAID^3^, THEN they should be provided concomitant treatment with a proton pump inhibitor.	n/a	n/a	n/a	n/a	n/a	9	No	8	No	Included	N1
10	IF oral pharmacological therapy is initiated to treat symptomatic osteoarthritis in an older adult, THEN paracetamol/acetaminophen should be the first drug used.	6	No	8	No	Uncertain	6	No	n/a	n/a	Excluded	
11	IF an older adult’s oral pharmacological therapy for symptomatic osteoarthritis is changed from acetaminophen to a different agent, THEN there should be evidence that they have trialled the maximum dose of paracetamol/acetaminophen.	7	No	6	No	Uncertain	n/a	n/a	6	No	Excluded	
12	IF an older adult is diagnosed with CNCP^2^, THEN provide multimodal, interprofessional treatment.	9	No	5	No	Uncertain	n/a	n/a	5.5	No	Excluded	
13	IF an older adult is diagnosed with CNCP^2^, THEN use base medication combined with as-needed medication.	7	No	8	No	Included	n/a	n/a	n/a	n/a	Included	G10
14	IF an older adult has CNCP^2^, THEN perform medication reviews regularly.	9	No	6.5	No	Included	n/a	n/a	n/a	n/a	Included	G2
15	IF an older adult has CNCP^2^, THEN monitor for adverse drug events.	9	No	6.5	No	Included	n/a	n/a	n/a	n/a	Included	G3
16	IF an older adult is being treated for CNCP^2^, THEN choose oral drugs.	8	No	7	No	Included	n/a	n/a	n/a	n/a	Included	G6
17	IF an older adult has localised CNCP^2^, THEN use topical drugs.	7	No	6.5	No	Included	n/a	n/a	n/a	n/a	Included	G8
18	IF an older adult is being treated for CNCP^2^, THEN choose topical drugs.	5.5	No	6.5	No	Uncertain	6.5	No	n/a	n/a	Excluded*	
19	IF an older adult has CNCP^2^, THEN use sustained release forms around the clock.	7	No	6.5	No	Included	n/a	n/a	n/a	n/a	Included	G9
20	IF an older adult with CNCP^2^ is treated using opioids, THEN use long-acting formulations.	8	No	8	No	Included	n/a	n/a	n/a	n/a	Included	O9
21	IF an older adult with CNCP^2^ is treated using opioids, THEN use short-acting opioids for breakthrough pain.	8	No	7	No	Included	n/a	n/a	n/a	n/a	Included	O10
22	IF an older adult with CNCP^2^ is treated using opioids, THEN they should be monitored for adverse drug events.	9	No	7	No	Included	n/a	n/a	n/a	n/a	Included	O4
23	IF an older adult with CNCP^2^ is eligible to be treated using opioids, THEN the potential for addiction should be assessed using validated tools prior to treatment initiation.	7	No	5	No	Uncertain	n/a	n/a	6	No	Excluded	
24	IF an older adult with CNCP^2^ is treated using opioids, THEN the potential for addiction should be monitored using validated tools.	7	No	5	No	Uncertain	n/a	n/a	5.5	No	Excluded	
N3	IF an older adult with CNCP^2^ has a higher addiction risk and requires opioids, THEN establish close monitoring and consider involving an addiction specialist.	n/a	n/a	n/a	n/a	n/a	8	No	5.5	No	Excluded	
25	IF an older adult with CNCP^2^ is treated using opioids, THEN their effects should be monitored.	9	No	8	No	Included	n/a	n/a	n/a	n/a	Included	O5
26	IF an older adult with CNCP^2^ is treated using opioids, THEN renal function should be monitored.	9	No	7	No	Included	n/a	n/a	n/a	n/a	Included	O8
27	IF an older adult with CNCP^2^ is treated using opioids, THEN use them as part of a multimodal approach.	9	No	5.5	No	Uncertain	n/a	n/a	6	No	Excluded	
28	IF an older adult with CNCP^2^ has tried all other options without success, THEN consider opioids.	8	No	7	No	Included	n/a	n/a	n/a	n/a	Included	O11
29.1	IF an older adult with CNCP^2^ and renal impairment is treated using opioids, THEN use an opioid without active metabolites.	9	No	7	No	Discussion	n/a	n/a	n/a	n/a	Excluded	
29.2	IF an older adult with CNCP^2^ and renal impairment is treated using opioids, THEN consider using…	n/a	n/a	n/a	n/a	n/a						
	…buprenorphine.	n/a	n/a	n/a	n/a	n/a	8	No	7	No	Included	O12
	…fentanyl.	n/a	n/a	n/a	n/a	n/a	7	No	6	No	Excluded	
	…hydromorphone.	n/a	n/a	n/a	n/a	n/a	8	No	8	No	Included	O12
	…oxycodone.	n/a	n/a	n/a	n/a	n/a	6.5	No	8	No	Included	O12
	…tapentadol.	n/a	n/a	n/a	n/a	n/a	7	No	5	No	Excluded	
30.1	IF an older adult with CNCP^2^ and hepatic impairment is treated using opioids, THEN use an opioid without active metabolites.	8	No	7	No	Discussion	n/a	n/a	n/a	n/a	Excluded	
30.2	IF an older adult with CNCP^2^ and hepatic impairment is treated using opioids, THEN consider hydromorphone.	n/a	n/a	n/a	n/a	n/a	8	No	7.5	No	Included	O16
31.1	IF an older adult with CNCP^2^ is treated using opioids, THEN avoid high doses.	6	No	5	No	Uncertain	n/a	n/a	n/a	n/a	Excluded	
31.2	IF an older adult with CNCP^2^ is started on opioids, THEN start with low doses (eg 0.1 mg morphine per kg body weight) and slowly titrate to the most effective tolerable dose.	n/a	n/a	n/a	n/a	n/a	8	No	8	No	Included	O2
32	IF an older adult with CNCP^2^ is treated using opioids, THEN other sedative drugs should be avoided.	8	No	6.5	No	Included	n/a	n/a	n/a	n/a	Included	O14
33	IF an older adult with CNCP^2^ is treated using opioids, THEN they should not receive benzodiazepines.	8	No	6	No	Uncertain	n/a	n/a	6.5	No	Included	O15
34	IF an older adult with CNCP^2^ requires opioids, THEN do not use tramadol.	7	No	7.5	No	Included	n/a	n/a	n/a	n/a	Included	O17
35	IF an older adult with CNCP^2^ requires opioids, THEN do not use morphine	4	No	6	No	Uncertain	4	No	6	No	Excluded	
N4	IF an older adult has CNCP^2^, THEN avoid long-term opioid therapy.	n/a	n/a	n/a	n/a	n/a	7	No	5	No	Excluded	
N5	IF an older adult with CNCP^2^ who has a neuropathic component requires opioids, THEN consider tapentadol.	n/a	n/a	n/a	n/a	n/a	7	No	6.5	No	Included	O18
N6	IF an older adult CNCP^1^ requires opioids, THEN avoid codeine.	n/a	n/a	n/a	n/a	n/a	8	No	8	No	Included	O13
36	IF an older adult has CNCP^2^, THEN do not use long-term NSAIDs^3^.	7	No	5	No	Uncertain	n/a	n/a	6	No	Excluded	
37	IF an older adult has CNCP^2^, THEN do not use NSAIDs^3^.	6	No	5	No	Uncertain	n/a	n/a	n/a	n/a	Excluded	
39	IF an older adult with CNCP^2^ has renal impairment, THEN do not use NSAIDs^3^.	9	No	8	No	Included	n/a	n/a	n/a	n/a	Included	N2
40	IF an older adult with CNCP^2^ has heart failure or other cardiovascular diseases, THEN do not use NSAIDs^3^.	8	No	7	No	Included	n/a	n/a	n/a	n/a	Included	N7
41	IF an older adult with CNCP^2^ has peptic ulcers or gastrointestinal bleeding, THEN do not use NSAIDs^3^.	9	No	7.5	No	Included	n/a	n/a	n/a	n/a	Included	N3
42	IF an older adult with CNCP^2^ has an H. pylori infection, THEN do not use NSAIDs^3^.	7	No	6	No	Uncertain	n/a	n/a	7	No	Included	N10
43	IF an older adult with CNCP^2^ is treated using NSAIDs^3^, THEN monitor for adverse drug events.	9	No	6.5	No	Included	n/a	n/a	n/a	n/a	Included	N4
44	IF an older adult with CNCP^2^ is treated using NSAIDs^3^, THEN do not use multiple NSAIDs^3^.	9	No	8	No	Included	n/a	n/a	n/a	n/a	Included	N6
45	IF an older adult with CNCP^2^ is treated using NSAIDs^3^, THEN do not combine with corticosteroids.	8	No	7	No	Included	n/a	n/a	n/a	n/a	Included	N8
46	IF an older adult with CNCP^2^ is treated using NSAIDs^3^, THEN do not combine with ACE-inhibitors^4^.	8	No	5.5	No	Uncertain	n/a	n/a	6	No	Excluded	
47	IF an older adult with CNCP^2^ requires NSAIDs^3^, THEN do not use indomethacin.	8	No	7.5	No	Included	n/a	n/a	n/a	n/a	Included	N9
48	IF an older adult with CNCP^2^ requires NSAIDs^3^, THEN do not use ketorolac.	7	No	8	No	Included	n/a	n/a	n/a	n/a	Included	N11
N7	IF an older adult with CNCP^2^ is being considered for treatment with an NSAID^3^, THEN consider testing for the CYP 2C9 genotype.	n/a	n/a	n/a	n/a	n/a	5	No	3	No	Excluded	
N8	IF an older adult with CNCP^2^ is being treated with an NSAID^3^ and qualifies for a proton pump inhibitor, THEN consider genotype testing for CYP 2C19.	n/a	n/a	n/a	n/a	n/a	5	No	3	No	Excluded	
49	IF an older adult with CNCP^2^ is treated using paracetamol, THEN do not exceed a dose of 4 g per day.	9	No	8	No	Included	n/a	n/a	n/a	n/a	Excluded^+^	
50	IF an older adult with CNCP^2^ is treated using paracetamol, THEN do not exceed a dose of 3 g per day.	9	No	8	No	Included	n/a	n/a	n/a	n/a	Included	P1
52.1	IF an older adult with CNCP^2^ and liver disease is treated using paracetamol, THEN adjust the dose.	9	No	8	No	Discussion	n/a	n/a	n/a	n/a	Excluded	
52.2	IF an older adult with CNCP^2^ and liver cirrhosis is treated using paracetamol/acetaminophen, THEN avoid daily doses above 2 g.	n/a	n/a	n/a	n/a	n/a	8	No	7	No	Included	P2
53	IF an older adult with CNCP^2^ has hepatopathologies, THEN do not use paracetamol.	7	No	7	No	Discussion	n/a	n/a	n/a	n/a	Excluded^#^	
54.1	IF an older adult with CNCP^2^ and chronic alcohol consumption is treated using paracetamol, THEN adjust the dose.	8	No	7	No	Discussion	n/a	n/a	n/a	n/a	Excluded	
54.2	IF an older adult with CNCP^2^ and chronic alcohol consumption is treated using paracetamol, THEN avoid daily doses above 2 g.	n/a	n/a	n/a	n/a	n/a	8	No	7	No	Included	P3
N9	IF an older adult has non-inflammatory CNCP^2^ and has tried paracetamol without achieving care goals, THEN consider metamizole.	n/a	n/a	n/a	n/a	n/a	7	No	7	No	Included	M3
N10	IF an older adult with CNCP^2^ is started on metamizole, THEN educate and monitor the patient for symptoms of agranulocytosis.	n/a	n/a	n/a	n/a	n/a	9	No	7	No	Included	M2
N11	IF an older adult has CNCP^2^ and low blood granulocyte counts or other agranulocytosis-inducing drugs, THEN avoid metamizole.	n/a	n/a	n/a	n/a	n/a	8.5	No	8	No	Included	M1
55	IF an older adult has neuropathic CNCP^2^, THEN consider co-analgesics.	9	No	7	No	Included	n/a	n/a	n/a	n/a	Included	C1
56.1	IF an older adult with CNCP^2^ is treated using TCAs^5^, THEN avoid high doses (10–100 mg)	8	No	7	No	Discussion	n/a	n/a	n/a	n/a	Excluded	
56.2	IF an older adult with CNCP^2^ is treated using TCAs^5^, THEN avoid high doses (eg no more than 10–25 mg of amitriptyline)	n/a	n/a	n/a	n/a	n/a	7	No	7	No	Included	C4
57	IF an older adult has CNCP^2^, THEN avoid TCAs^5^.	7.5	No	7	No	Included	n/a	n/a	n/a	n/a	Included	C3
58.1	IF an older adult with CNCP^2^ has closed-angle glaucoma, benign prostate hyperplasia, urinary retention, constipation, cardiovascular diseases or severe hepatic disease, THEN avoid TCAs^5^.	9	No	6	No	Uncertain	n/a	n/a	n/a	n/a	Excluded	
58.2	IF an older adult with CNCP^2^ has closed-angle glaucoma, benign prostate hyperplasia, urinary retention, constipation, cardiovascular diseases or severe hepatic disease, THEN avoid TCAs^5^.	n/a	n/a	n/a	n/a	n/a	8	No	7	No	Included	C2
59	IF an older adult with CNCP^2^ requires TCAs^5^, THEN use nortriptyline or desipramine.	5	No	5.5	No	Uncertain	6	No	7	No	Excluded	
60	IF an older adult with CNCP^2^ requires an anticonvulsant, THEN do not use carbamazepine.	6	No	7	No	Included	6.5	No	n/a	n/a	Included	C5
N12	IF an older adult with CNCP^2^ is being considered for treatment with carbamazepine, THEN consider HLA genotype testing.	n/a	n/a	n/a	n/a	n/a	6.5	No	5	No	Excluded	
61	IF an older adult has CNCP^2^, THEN do not treat it using gabapentinoids.	5	No	5	No	Uncertain	4.5	No	5	No	Excluded	
62	IF an older adult has CNCP^2^, THEN do not treat it using SSRIs^6^.	5	Yes	7	No	Uncertain	5.5	No	n/a	n/a	Excluded	
63	IF an older adult has CNCP^2^, THEN do not treat it using SNRIs^7^.	4	No	5	No	Uncertain	4	No	5	No	Excluded	
64	IF an elder with CNCP^2^ requires an SNRI^7^, THEN prefer Duloxetine to Venlafaxine.	6.5	No	7	No	Included	n/a	n/a	n/a	n/a	Included	C6
N13	IF an older adult with CNCP^2^ is being considered for treatment with oxycodone, codeine, tramadol or a TCA^5^, THEN consider genotype testing for CYP 2D6.	n/a	n/a	n/a	n/a	n/a	6	No	5	No	Excluded	

**Notes**: ^1^ Dis, Disagreement as defined by the RAND/UCLA appropriateness method; ^2^ CNCP, Chronic non-cancer pain; ^3^ NSAIDs, Non-steroidal anti-inflammatory drugs; ^4^ ACE-inhibitors, angiotensin-converting enzyme inhibitors; ^5^ TCAs, tri-cyclic anti-depressants; ^6^ SSRIs, selective serotonin reuptake inhibitors; ^7^ SNRIs, serotonin noradrenalin reuptake inhibitors. * Also see the corresponding results section. Excluded because of similarities to QI 17 and expert recommendations. ^+^ Excluded because experts preferred QI 49. ^#^ Also see the corresponding results section. Excluded during focus group discussion because of expert consensus. QIs with a decimal place (eg 2.1 and 2.2) were adapted during the focus group discussions.A green background indicates inclusion, yellow indicates uncertain ratings, and red indicates exclusion. New numbers were given to all the QIs retained and can be seen in Table 3.

**Table 3:** The Final Set of Quality Indicators (QIs) for the Pharmacological Management of Chronic Non-Cancer Pain in Older Adult Inpatients

No.	Quality indicator	Validity	Feasibility	Priorities
General drug therapy				
G1	IF an older adult has CNCP^1^, THEN define care goals with the patient that focus on quality of life and functionality.	9	7	5
G2	IF an older adult has CNCP^1^, THEN perform medication reviews regularly.	9	6.5	2
G3	IF an older adult has CNCP^1^, THEN monitor for adverse drug events.	9	6.5	2
G4	IF an older adult is being treated for CNCP^1^, THEN they should be assessed for a response within 1 month.	8	7	2
G5	IF an older adult has a new diagnosis of CNCP^1^, THEN pharmacological treatment should be provided.	7	8	2
G6	IF an older adult is being treated for CNCP^1^, THEN choose oral drugs.	8	7	0
G7	IF an older adult with CNCP^1^presents with acute pain (score ≥ 6 on a scale of 0–10 or a similar quantifiable measure on an assessment tool for cognitively impaired patients), THEN pain treatment should be adjusted if aligned with care goals.	8	8	0
G8	IF an older adult has localised CNCP^1^, THEN use topical drugs.	7	6.5	0
G9	IF an older adult has CNCP^1^, THEN use sustained release forms around the clock.	7	6.5	0
G10	IF an older adult is diagnosed with CNCP^1^, THEN use base medication combined with as-needed medication.	7	8	0
Opioids				
O1	IF an older adult with CNCP^1^ starts a new opioid therapy or if the opioid dose is changed, the efficacy and side effects should be closely monitored and evaluated no later than 1 week after starting/changing therapy.	9	7.5	3
O2	IF an older adult with CNCP^1^ is started on opioids, THEN start with low doses (eg 0.1 mg morphine per kg body weight) and slowly titrate to the most effective tolerable dose.	8	8	2
O3	IF an older adult with CNCP^1^ starts opioid therapy, THEN evaluate after no more than 3 months whether the therapy significantly improves the patient’s quality of life.	9	7	1
O4	IF an older adult with CNCP^1^ is treated using opioids, THEN they should be monitored for adverse drug events.	9	7	1
O5	IF an older adult with CNCP^1^ is treated using opioids, THEN their effects should be monitored.	9	8	1
O6	IF an older adult with CNCP^1^ is treated using opioids, THEN they should be offered a bowel regimen or medical records should document the potential for constipation or explain why bowel treatment is not needed.	9	7	0
O7	IF an older adult with CNCP^1^ requires analgesia, THEN pethidine/meperidine should not be used.	9	9	0
O8	IF an older adult with CNCP^1^ is treated using opioids, THEN renal function should be monitored.	9	7	0
O9	IF an older adult with CNCP^1^ is treated using opioids, THEN use long-acting formulations.	8	8	0
O10	IF an older adult with CNCP^1^ is treated using opioids, THEN use short-acting opioids for breakthrough pain.	8	7	0
O11	IF an older adult with CNCP^1^ has tried all other options without success, THEN consider opioids.	8	7	0
O12	IF an older adult with CNCP^1^ and renal impairment is treated using opioids, THEN consider using buprenorphine, hydromorphone or oxycodone.	8	8	0
O13	IF an older adult with CNCP^1^ requires opioids, THEN avoid codeine.	8	8	0
O14	IF an older adult with CNCP^1^ is treated using opioids, THEN other sedative drugs should be avoided.	8	6.5	0
O15	IF an older adult with CNCP^1^ is treated using opioids, THEN they should not receive benzodiazepines.	8	6.5	0
O16	IF an older adult with CNCP^1^ and hepatic impairment is treated using opioids, THEN consider hydromorphone.	8	7.5	0
O17	IF an older adult with CNCP^1^ requires opioids, THEN do not use tramadol.	7	7	0
O18	IF an older adult with CNCP^1^ who has a neuropathic component requires opioids, THEN consider tapentadol.	7	6.5	0
Non-steroidal anti-inflammatory drugs (NSAIDs)				
N1	IF an older adult with CNCP^1^ - has a history of peptic ulcer disease or gastrointestinal bleeding and/or - currently uses antithrombotics, anticoagulants, corticosteroids or SSRIs^2^ AND they are treated using a cyclooxygenase-nonselective NSAID^3^, THEN they should be provided concomitant treatment with a proton pump inhibitor.	9	8	7
N2	IF an older adult with CNCP^1^ has renal impairment, THEN do not use NSAIDs^3^.	9	8	3
N3	IF an older adult with CNCP^1^ has peptic ulcers or gastrointestinal bleeding, THEN do not use NSAIDs^3^.	9	7.5	1
N4	IF an older adult with CNCP^1^ is treated using NSAIDs^3^, THEN monitor for adverse drug events.	9	6.5	0
N5	IF an older adult has been prescribed a cyclooxygenase-nonselective NSAID^3^ for the treatment of CNCP^1^, THEN medical records should indicate whether they have a history of peptic ulcer disease and, if they do, justification of NSAID^3^ use should be documented.	9	7	0
N6	IF an older adult with CNCP^1^ is treated using NSAIDs^3^, THEN do not use multiple NSAIDs^3^.	9	8	0
N7	IF an older adult with CNCP^1^ has heart failure or other cardiovascular diseases, THEN do not use NSAIDs^3^.	8	7	0
N8	IF an older adult with CNCP^1^ is treated using NSAIDs^3^, THEN do not combine with corticosteroids.	8	7	0
N9	IF an older adult with CNCP^1^ requires NSAIDs^3^, THEN do not use indomethacin.	8	7.5	0
N10	IF an older adult with CNCP^1^ has an H. pylori infection, THEN do not use NSAIDs^3^.	7	7	0
N11	IF an older adult with CNCP^1^ requires NSAIDs^3^, THEN do not use ketorolac.	7	8	0
Paracetamol				
P1	IF an older adult with CNCP^1^ is treated using paracetamol, THEN do not exceed a dose of 3 g per day.	9	8	5
P2	IF an older adult with CNCP^1^ and liver cirrhosis is treated using paracetamol, THEN avoid daily doses above 2 g.	8	7	3
P3	IF an older adult with CNCP^1^ and chronic alcohol consumption is treated using paracetamol, THEN avoid daily doses above 2 g.	8	7	2
Metamizole				
M1	IF an older adult has CNCP^1^ and low blood granulocyte counts or other agranulocytosis-inducing drugs, THEN avoid metamizole.	8.5	8	6
M2	IF an older adult with CNCP^1^ is started on metamizole, THEN educate and monitor the patient for symptoms of agranulocytosis.	9	7	5
M3	IF an older adult has non-inflammatory CNCP^1^ and has tried paracetamol without achieving care goals, THEN consider metamizole.	7	7	3
Co-analgesics				
C1	IF an older adult has neuropathic CNCP^1^, THEN consider co-analgesics.	9	7	8
C2	IF an older adult with CNCP^1^ has closed-angle glaucoma, benign prostate hyperplasia, urinary retention, constipation, cardiovascular diseases or severe hepatic disease, THEN avoid TCAs^4^.	8	7	2
C3	IF an older adult has CNCP^1^, THEN avoid TCAs^4^.	7.5	7	1
C4	IF an older adult with CNCP^1^ is treated using TCAs^4^, THEN avoid high doses (eg no more than 10–25 mg of amitriptyline).	7	7	1
C5	IF an older adult with CNCP^1^ requires an anticonvulsant, THEN do not use carbamazepine.	6.5	7	0
C6	IF an elder with CNCP^1^ requires an SNRI^5^, THEN prefer Duloxetine to Venlafaxine.	6.5	7	0

**Notes**: The table provides the final median face validity and feasibility scores and the number of times the indicator was mentioned as a priority. Indicators are ordered by priority, validity and then feasibility. The number of priorities does not add up to the total number of experts, as some QIs selected as priorities were excluded due to either insufficient median face validity or feasibility ratings.

**Abbriviations**: CNCP, chronic non-cancer pain; SSRI, selective serotonin reuptake inhibitor; NSAIDs, non-steroidal anti-inflammatory drugs; TCAs, tri-cyclic anti-depressants; SNRIs, serotonin noradrenalin reuptake inhibitors.

### Focus Group

Eighteen of our 22 experts (81%) agreed to participate in one of our three focus group discussions; however, mostly due to clinical emergencies, only 9 (50%) of them actually did so. The experts discussed the “uncertain” QIs, the QIs with suggested improvements and the new QIs proposed by the experts in round one. As a result, 11 QIs were modified before being rated again, and 13 new QIs were proposed for rating in round two. The experts deemed one QI to be inappropriate, and we excluded it. Table 2 provides an overview of the changes made.

Regarding general drug therapy, the experts discussed when patients should have their medication re-evaluated and the value of numerical rating scales. QI 2 advised that a patient’s medication should be re-evaluated after every 3–6 months of therapy. In their discussions, the experts agreed that this period was too long. The consensus was that treatment re-evaluations would ideally occur as soon as 1 week after treatment initiation or change. As this would decrease the QI’s feasibility, the experts agreed to the wording “within 1 month”. In their opinion, this QI should emphasise that if an earlier evaluation were possible, then it should be carried out. The experts discussed the value of numerical rating scales at length (*eg* 0–10, where 0 is no pain and 10 is the worst pain imaginable). Overall, their consensus was that numerical rating scales were suboptimal for evaluating the adequacy of an ongoing treatment. This led to the development of QI N1, specifying that treatment goals should align with quality of life and functionality. The majority of experts nevertheless believed that numerical rating scales might be more suitable to evaluate treatment efficacy in acute on chronic pain (eg patients with a fracture and chronic low back pain), aligning with standard evaluations of acute pain management. This resulted in a modified version of QI 3. However, some experts voiced the opinion that acute CNCP should not lead to an immediate change in pain medication but rather to the initiation of a careful clinical evaluation of the patient. Experts also agreed that many older adults find numerical rating scales problematic, particularly those with a cognitive impairment.

When discussing the QIs on opioids, the experts focussed on therapeutic monitoring and the risks of addiction. Regarding opioid initiation, the experts stressed the importance of distinguishing safety and efficacy. Safety should be evaluated earlier—within one week, according to QI 5.2. Experts also underlined the importance of nurses monitoring the patient. Efficacy, on the other hand, should be measured with improvements in quality of life. The experts emphasised that if no improvements occurred, opioid therapy should be stopped. They also acknowledged the importance of screening for and monitoring addiction to opioids. However, they doubted the feasibility of implementing this widely, especially in a European context. They argued that clinicians had limited time and lacked the necessary knowledge about screening tools and how to interpret results.

The experts noted that a scarcity of evidence made discussing QIs on metamizole challenging; only a few countries use this active substance. There was discussion about whether metamizole should be a first-line drug, comparable to paracetamol, but due to the lack of evidence, especially on efficacy and safety, they agreed to recommend using metamizole solely in cases of non-inflammatory CNCP where paracetamol is insufficiently effective. In doing so, the experts acknowledged the increased risk of agranulocytosis when using metamizole, and they proposed two QIs as risk-reduction strategies.

The experts also discussed pharmacogenetic testing’s value in CNCP management. While most agreed that it might be relevant in some cases, they had doubts about the cost–benefit analysis and healthcare professionals’ ability to interpret the testing’s results. Some participants also voiced concerns that the pharmacogenetic testing of polymedicated older adults with CNCP might be inferior to phenotyping as drug–drug, drug–gene and drug–disease interactions become too complex to predict.

### Second Round

Nineteen (86%) experts participated in the second round, rating the “uncertain”, modified and new QIs and prioritising them all according to their relevance. These ratings led to a final set of 51 QIs meeting our pre-specified inclusion criteria. QI 18 (prefer topical drugs) received a sufficiently high median rating to be included; however, multiple experts stated that it was less valid than QI 17 (prefer topical drugs for localised pain). Due to their similarity, we followed their recommendation and excluded it. All the median QI ratings are shown in Table 2. Twenty-three QIs received at least one priority rating. The final set of QIs, ordered by priority, face validity and then feasibility, is shown in Table 3. It consists of 10 QIs for general drug therapy, 18 for opioids, 11 for NSAIDs, 3 for paracetamol, 3 for metamizole and 5 for co-analgesics.

## Discussion

This RAM Delphi study aimed to reach a consensus on the face validity and feasibility of a set of potential QIs for the pharmacological management of CNCP in older adult inpatients. After two written rounds rating the QIs and three focus group discussions between them, our experts included a total of 51 QIs, of which 23 were given a priority rating. To the best of our knowledge, this is the first set of expert-validated QIs prepared for the pharmacological management of CNCP in older adult inpatients.

We chose a RAM Delphi study approach based on a systematic literature search because this is a recommended process for developing a set of QIs.^18^ The Delphi method allows experts to construct a shared clinical reality based on an aggregated scientific evidence base.^22^ It is particularly useful because its anonymous rating rounds ensure that every expert’s opinions are heard, reducing the risk of a few dominant voices overpowering others. Having focus group discussions between the anonymous written rounds facilitates the exchange of opinions needed to reach a consensus.^24^ Predefined inclusion and exclusion criteria for QIs, set as per the RAM recommendations, provided the basis for a rigorous evaluation of the QIs. Finally, combining the results of a systematic literature search with input from experts working clinically with older adults enabled the development of a set of QIs that were both evidence-based and relevant to practice. Delphi study methodologies are not above critique, however, with some authors arguing that they are neither reliable nor valid enough.^25^,^26^ The RAM acknowledges this, stating that Delphi studies are more reliable and valid when there is a sound evidence base and when the items rated are objective.^23^ Keeney et al^27^ also indicated that judging the constructivist Delphi method using positivist criteria (reliability and validity) may be inappropriate. Instead they suggested quality criteria such as transferability, credibility, applicability and confirmability.^27^

The set of QIs developed in this study could be used in different ways. They could help to standardise the pharmacological care delivered to older adult inpatients with CNCP^18^ or could be used as trigger tools integrated into an electronic algorithm to flag patients at an elevated risk of MRPs or to suggest targeted medication reviews.^19^ To this end, further research validating our set of QIs in clinical practice seems warranted. Feedback on the set’s clinical applicability and completeness would have to be collected systematically: the set of QIs may be too big to be useful in clinical practice or may lack depth to support decision-making. Indeed, the set of QIs prepared involved no patient input, and some QIs may not adequately reflect aspects of the quality of care that are most relevant to them. Extrapolation to other situations may also be needed, as some of the QIs could also be applied to outpatient care. Another point to consider is new developments. As technologies evolve, such as pharmacogenetic testing, they may become more affordable and available, necessitating their integration into this set of QIs. It is also worth noting that important insights can be gained through a thorough examination of the patient’s medical and family history. These can serve as accessible alternatives or complementary approaches.

Although most of the QIs excluded from our final set were considered valid—sometimes highly valid—our experts doubted that current healthcare systems would be able to implement them, making them infeasible. One specific example of this was QI 12, which recommends multimodal treatments. This concept is reflected in most clinical guidelines.^12^,^28^,^29^ Even though our experts rated this QI with a median of 9 and gave it two priority ratings, its feasibility was deemed too low. Reasons for this included too few specialised healthcare professionals and health insurance reimbursement issues for the institutions concerned.

Some QIs included in this set may prove controversial. For instance, QI 1 recommends prescribing pharmacological treatments to all older adult inpatients with CNCP. Many guidelines recommend that pharmacological treatments should only be second-line treatments.^12^,^28^,^29^ On the contrary, however, our experts stated that pharmacological therapy should at least be available as a first-line safety net, and they noted that most older adult inpatients with CNCP were currently treated pharmacologically anyway, despite the guidelines.

Other QIs involving some uncertainties included those for metamizole. Metamizole is only licensed in a few countries, but it is used a lot in Switzerland, Austria and Germany, particularly for older adults.^30^,^31^ Although there is some evidence that metamizole may indeed be more tolerable than NSAIDS and just as efficient,^32^ many healthcare professionals still fear agranulocytosis, a very rare but potentially lethal adverse drug reaction.^33^ As metamizole is only used in a handful of countries, evidence on its long-term use remains limited.^34^ Evaluations of recommendations for its use, therefore, often rely on personal clinical experience. With new evidence on metamizole emerging, for example, regarding drug–drug interactions and efficacy, the QIs that we have proposed here may need updating.^35^,^36^

## Limitations

Although this Delphi study was performed following a systematic literature search and conducted using the RAM’s rigorous methodology, it had some limitations. First, the quality of the consensus reached in any Delphi study relies on the quality of the available evidence and the panel members’ clinical expertise. Because CNCP in older adult inpatients has been poorly studied, the evidence base may be too weak to formulate robust QIs. However, as the rate of retention of the QIs from the literature was high, we believe that the selection presented to the experts was appropriate. Second, participation in the focus group discussions was low. Although we tried to make participating in a focus group as easy as possible, many experts failed to attend. We nevertheless fully informed every expert about the discussions, whether they had attended one or not, and allowed them to give full written input on our suggested QIs in both rounds. We believe, therefore, that even if some experts did not participate in the focus group discussions, they had sufficient possibilities to intervene. Third, the QIs present here are not generalisable, particularly regarding their feasibility. Our experts were predominantly from German-speaking countries, and their recommendations may reflect the clinical practices in those countries. The feasibility of QIs, in particular, may also vary between institutions.

## Conclusion

This two-round Research and Development Corporation (RAND) and University of California at Los Angeles (UCLA) Appropriateness Method Delphi study involved three focus groups and 19 experts rating potential quality indicators (QIs) for the pharmacological management of chronic non-cancer pain (CNCP) in older adult inpatients. Our experts included 51 QIs in the final set. This is the first expert-validated set of QIs developed out of a systematic search of the relevant literature, and it could help to support the standardisation of the care provided to older adult inpatients with CNCP, increasing the overall quality of the care delivered to them. These QIs could also be used as trigger tools to prioritise patients for interventions. To this end and to generalise the QIs to other settings, our QIs may still need to be adapted to local clinical practices and imperatives, and they require further clinical validation using real-world data.

## Abbreviations

CNCP, chronic non-cancer pain; CREDES, Conducting and Reporting of Delphi Studies; IPRAS, Interpercentile Range Adjusted for Symmetry required for disagreement; NSAID, non-steroidal anti-inflammatory drugs; QI, quality indicator; RAM, RAND/UCLA Appropriateness Method; RAND, Research and Development Corporation; UCLA, University of California, Los Angeles.
